# Molecular Pathogenesis, Tumor Microenvironment and Health Disparities in Select Pediatric Solid Tumors: An Integrative Narrative Review

**DOI:** 10.3390/diseases14090307

**Published:** 2026-08-25

**Authors:** MiaSara Pérez-Salvá, Carolyn M. Ruiz-Pérez, Alondra Veloz-Bonilla, Rocío K. Rivera-Valentín

**Affiliations:** 1 School of Medicine, University of Puerto Rico, Medical Sciences Campus, San Juan 00935, Puerto Rico; miasara.perez@upr.edu; 2 Division of Clinical and Traslational Cancer Research, University of Puerto Rico, Comprehensive Cancer Center, San Juan 00935, Puerto Rico; cruiz@cccupr.org (C.M.R.-P.);

**Keywords:** pediatric oncology, disparities, pediatric solid tumors, molecular pathogenesis, tumor microenvironment, therapeutic resistance, health equity, molecular diagnosis, precision medicine

## Abstract

Background/Objectives: Pediatric solid tumors (PST) are a biologically distinct group of malignancies whose developmental origins and molecular drivers differ substantially from those of adult cancers, with direct implications for therapeutic strategy and clinical outcome. This review synthesizes current evidence on molecular pathogenesis, tumor microenvironment biology, and the structural conditions that shape access to care across select PST. Methods: A narrative review of peer-reviewed literature was conducted primarily using PubMed, supplemented by Google Scholar, covering publications from 2000 to 2025. Tumor types were selected based on their prevalence in the pediatric population and the availability of evidence addressing both molecular features and health disparities. Body: Across eight tumor types (neuroblastoma, Ewing sarcoma, pediatric brain tumors, rhabdomyosarcoma, Wilms tumor, retinoblastoma, osteosarcoma, and chondrosarcoma), recurrent molecular alterations including MYCN amplification, EWS-FLI1 fusions, PAX-FOXO1 rearrangements and IDH 1/2 mutations emerge as central determinants of disease behavior and eligibility for treatment. The tumor microenvironment manifests as a shared mediator of immune exclusion and therapeutic resistance across tumor types, with, but not limited to, tumor-associated macrophages, myeloid-derived suppressor cells, and checkpoint molecule expression, identified as recurrent features influencing treatment response. Immunotherapeutic strategies have shown variable efficacy across PST, with the most consistent clinical benefit established in neuroblastoma. A critical and underappreciated pattern stands out across tumor types: children carrying the most aggressive molecular subtypes are disproportionately those with the least access to therapies those subtypes demand, emphasizing an overlap of biological and structural disadvantage that is also amplified in low- and middle-income countries, where late-stage presentation, treatment abandonment and limited access to molecular diagnostics compound the biological disadvantage. Conclusions: Within the eight PST reviewed, the most aggressive molecular subtypes and the greatest structural disadvantages converge in the same children; those carrying MYCN amplification, PAX-FOXO1 fusions, or EWS-FLI1 fusions are disproportionately those with the least access to the therapies their biology demands. Genomic and immunologic advances will only reach their full clinical potential when paired with inclusive trial data, diversified genomic databases, and most importantly, equitable access to biomarker-specialized therapies across all populations.

## 1. Introduction

Pediatric solid tumors (PST) encompass a diverse group of malignancies that arise in both children and adolescents, accounting for approximately 30% of all childhood cancers [1]. These tumors include neuroblastoma, brain tumors, malignant bone tumors, and sarcomas, each presenting distinct clinical and mechanistic profiles. Unlike adult solid tumors, pediatric tumors exhibit unique biological features closely linked to developmental processes and defined by specific molecular alterations, which, in turn, influence their response to treatment [2]. Despite significant advances in multimodal therapy, PST is a leading cause of cancer-related mortality in children and adolescents, with an estimated five-year survival rate that varies widely across tumor types and patient populations [3].

Recent genomic and epidemiological studies have provided valuable insights into the pathogenesis and heterogeneity of PST. Integrative clinical sequencing applied to these population groups with refractory or relapsed malignancies has demonstrated that a meaningful proportion harbor pathogenic variants in cancer-predisposing genes, demonstrating the importance of inherited risk and supporting the incorporation of genomic screening into clinical evaluation [4]. Advances in next-generation sequencing and molecular diagnostics have also enabled earlier detection and improved risk stratification, though the translation of identified molecular targets into approved therapies for pediatric solid tumors is still limited [4,5]. However, systemic and socioeconomic challenges continue to impede the implementation of these discoveries into clinical practice, particularly in resource-limited settings where access to advanced diagnostics and targeted therapies is restricted.

**Figure 1:**
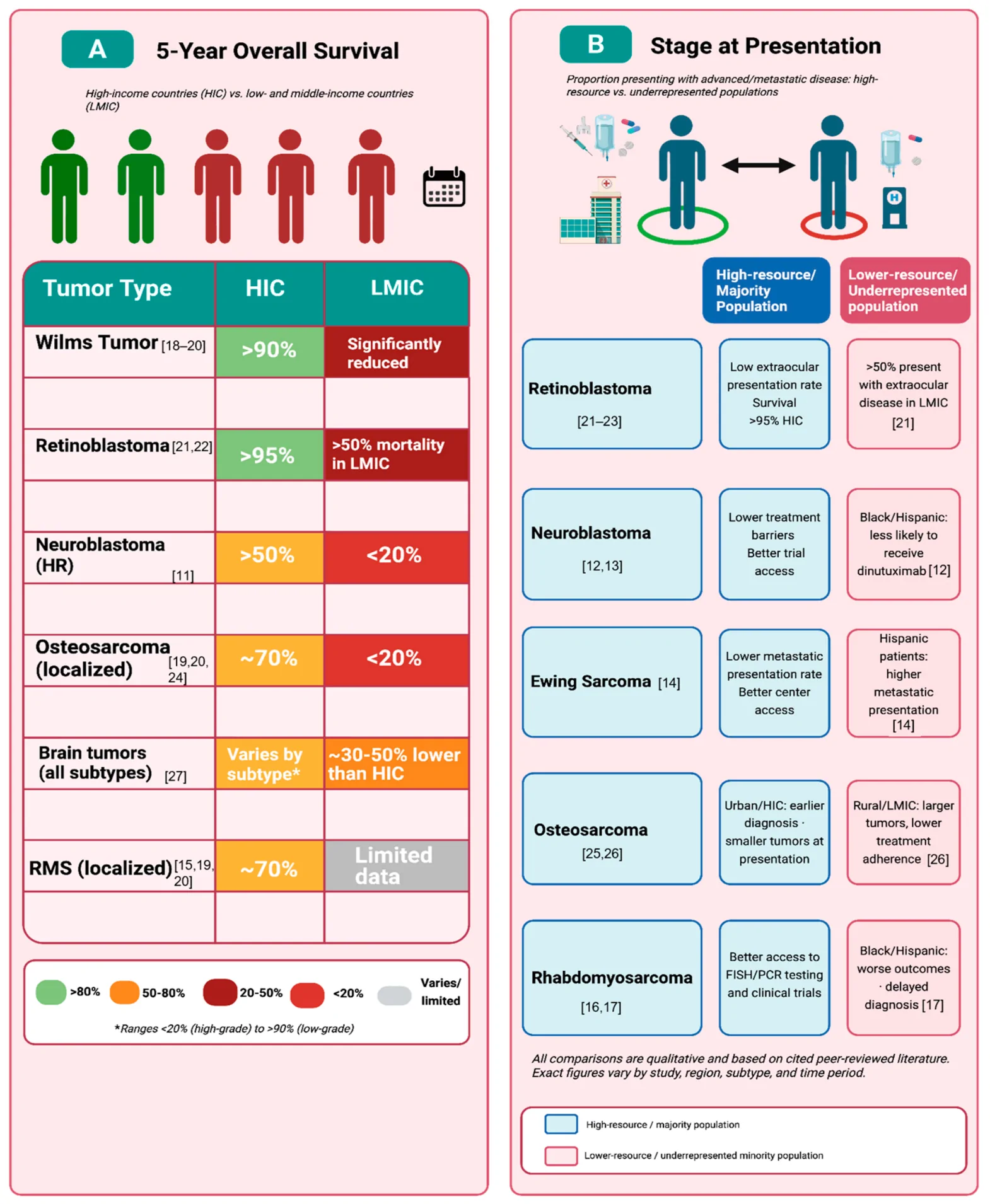
Five-year overall survival and outcome disparities across select pediatric solid tumors by healthcare setting and patient demographics. (**A**) Five-year overall survival rates comparing high-income countries (HIC) versus low- and middle-income countries (LMIC), derived from cited peer-reviewed literature. Brain tumor survival varies substantially by subtype. (**B**) Documented disparities in outcomes and access to care comparing high-resource versus lower-resource or underrepresented minority populations. HIC, high-income countries; LMIC, low- and middle-income countries; RMS, rhabdomyosarcoma; OS, osteosarcoma; RB, retinoblastoma; WT, Wilms tumor; ES, Ewing sarcoma [11,12,13,14,15,16,17,18,19,20,21,22,23,24,25,26,27]. Created in BioRender. Pérez salvá, M. (2026) https://BioRender.com/q1d1ao2.

Disparities in outcomes for PST remain pronounced, particularly among Hispanic and Black pediatric populations. These patterns point to structural inequities that shape healthcare access and delivery, influencing diagnostic delays, treatment initiation, and the availability of comprehensive oncologic and supportive services [6,7]. Studies have shown that these populations often experience delays in diagnosis and reduced participation in clinical trials compared to non-Hispanic White populations [8,9]. Adolescents with solid tumors also face substantial challenges related to transitional care and psychosocial support, leading to poorer outcomes and long-term survivorship compared to younger pediatric patients [10]. These difficulties arise because adolescents occupy a developmental and clinical intersection: they are often too old for the highly coordinated, family-centered pediatric model yet not fully integrated into adult oncology systems that assume greater independence. As a result, gaps emerge in diagnosis, support, and continuity of care at a critical stage of development, shaping both immediate treatment experiences and long-term survivorship. It is imperative to note that these challenges are not confined to the United States. Across low- and middle-income countries, the picture is even more stark- five-year survival rates for several PST fall below 20% in sub-Saharan African settings, treatment abandonment rates exceeding 50% have been reported in some regions [11,12], and even tumors with highly favorable outcomes in high-income settings, such as Wilms tumor and retinoblastoma, carry a substantially worse prognosis where early detection and standardized protocols remain out of reach [13]. This review incorporates international data where available, recognizing that advances in precision oncology are not used to their full potential without parallel investments in equitable healthcare delivery worldwide. Survival outcomes across tumor types and healthcare settings are summarized in Figure 1.

Amid these disparities, which highlight persistent social and clinical barriers to optimal care, there is also an important intersection with biological constraints that complicate disease management. Understanding the tumor microenvironment is therefore essential, as its immunological and molecular dynamics directly influence several factors ranging from treatment resistance to the feasibility of emerging therapeutic strategies. It shapes tumor progression and therapeutic response by regulating immune cell infiltration and the balance between immune activation and suppression. Immunotherapeutic strategies that directly modulate antitumor immunity, including immune checkpoint blockade, chimeric antigen receptor (CAR) T cell therapy, and adoptive transfer of tumor-infiltrating lymphocytes, have been explored in PST, with variable efficacy [28]. With the exception of neuroblastoma, where dinutuximab targeting GD2 has demonstrated consistent clinical benefit, immune-based strategies have not yet achieved durable response across PST, reflecting the shared immunosuppressive architecture of their tumor microenvironments [29]. However, mechanisms of immune evasion and tumor antigen heterogeneity, without disregarding potential toxicity, remain key challenges in integrating immunotherapy into standard treatment regimens [29]. Ongoing preclinical studies and early-phase clinical trials are investigating immunotherapy-relevant biomarkers and novel immune targets to improve patient stratification and therapeutic responsiveness [30]. Heterogeneity in tumor immune landscapes, however, continues to complicate the development of standardized treatment strategies across PST [30]. The challenges described above continue to delay clinical translation. Diverging hypotheses involving genomic approaches such as epigenetics, as well as efforts focused on inflammatory signaling and environmental exposures, continue to shape ongoing research directions [31].

This review provides an integrative overview of select PSTs, examining recent progress alongside enduring obstacles that continue to shape patient outcomes. By connecting tumor biology with structural inequities in care and evolving therapeutic paradigms, this work emphasizes the importance of sustained interdisciplinary collaboration to address unmet needs in pediatric oncology research and clinical practice, particularly for historically underserved populations. The synthesis of molecular risk factors with documented structural access barriers across three high-incidence tumor types (neuroblastoma, Ewing sarcoma, and rhabdomyosarcoma) represents a contribution not previously consolidated in the PST review literature.

## 2. Neuroblastoma

Neuroblastoma, recognized as the most common extracranial solid malignancy in children, accounts for more than 650 cases annually in the United States and arises from neural crest–derived sympathoadrenal progenitor cells, most frequently originating in the adrenal medulla or along the sympathetic chain, with peak incidence occurring in children younger than five years of age [14]. Its clinical course spans a broad spectrum that ranges from spontaneous regression in infants to aggressive metastatic disease in older patients; a spectrum determined by the interplay between tumor genetics and host biology in ways that remain incompletely understood [32].

Clinical presentation may include abdominal distension, localized pain, weight loss, fatigue, or manifestations of paraneoplastic syndromes such as opsoclonus–myoclonus–ataxia, with diagnostic evaluation relying on cross-sectional imaging, including CT and MRI, as well as MIBG scintigraphy, followed by histopathological confirmation through biopsy and biochemical assessment of catecholamine metabolites in urine or serum. Disease classification is established using the International Neuroblastoma Staging System (INSS) or the more recent International Neuroblastoma Risk Group (INRG) framework, both of which integrate clinical and genomic parameters to refine risk stratification [14,15].

At the molecular level, neuroblastoma is defined by recurrent genomic alterations that distinguish clinically relevant subgroups, including amplification of *MYCN*, present in approximately 20–25% of cases and strongly associated with high-risk disease and unfavorable outcomes, as well as additional chromosomal abnormalities such as deletions of 1p and 11q, gain of 17q, and other segmental changes that add to disease classification [32]. Susceptibility is also influenced by germline variation, exemplified by regulatory variants in the *LMO1* gene that promote an adrenergic cellular phenotype and increase disease risk [33,34].

Differences in clinical outcomes are shaped not only by tumor-intrinsic features but also by broader structural and demographic conditions, as marked disparities have been reported across racial and ethnic groups, with Black and Hispanic children demonstrating lower overall and event-free survival compared to non-Hispanic White patients even after accounting for treatment intensity and risk classification [8,9]. These patterns clearly show inequities in access to early diagnosis, specialized care, and clinical trial participation, and are illustrated by observations that Hispanic children in the United States are more likely to present with advanced-stage disease and less likely to undergo complete surgical resection or receive immunotherapy, findings that correlate with poorer outcomes [15].

**Figure 2:**
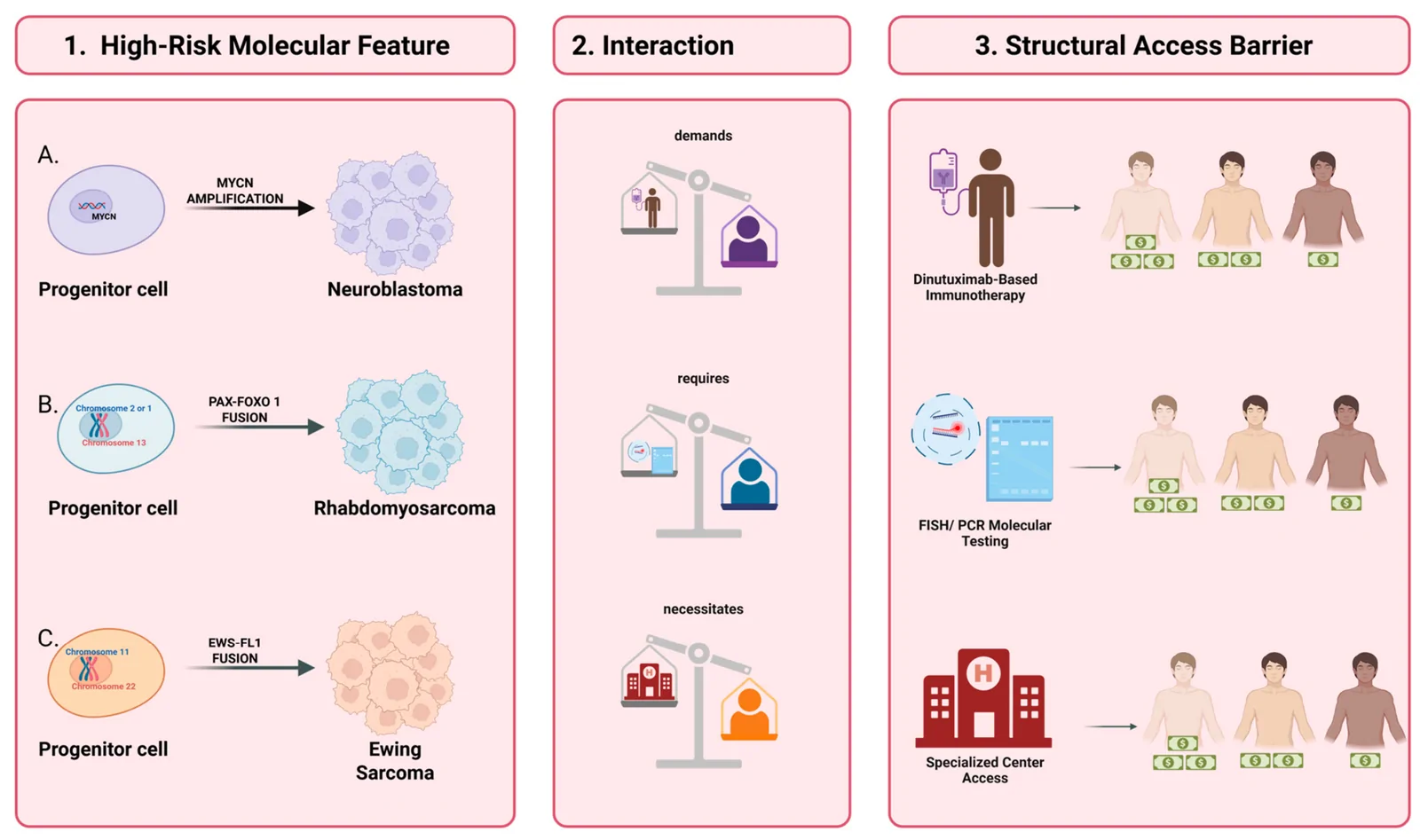
**Convergence of molecular risk and structural disadvantage in select pediatric solid tumors.** Each row presents a high-risk molecular feature paired with the specific diagnostic or therapeutic modality it demands, for which access is documented to be unequally distributed across racial, ethnic, or socioeconomic lines. NB, neuroblastoma; RMS, rhabdomyosarcoma; ES, Ewing sarcoma; FISH, fluorescence in situ hybridization; PCR, polymerase chain reaction. Created in BioRender. Veloz, A. (2026) https://BioRender.com/vhivu30.

Socioeconomic conditions also influence disease trajectories, as children from low-income households or those covered by public insurance frequently encounter delays in treatment initiation, increased risk of therapy-related toxicity, and challenges adhering to demanding multimodal regimens [8,9]. Geographic factors add another dimension, particularly in rural and underserved regions where access to high-volume pediatric oncology centers is limited, thereby reducing the likelihood of receiving guideline-concordant care or participating in clinical studies. At the level of genomic research, disparities remain evident because populations of non-European ancestry are significantly underrepresented in neuroblastoma genetic studies, limiting the applicability of precision-based approaches, while the lack of diversity in tumor and germline datasets can obscure ancestry-specific variants and therapeutic vulnerabilities, limiting unequal access to advances in biologically informed treatment [33]. Critically, children with MYCN-amplified high-risk neuroblastoma—the subgroup with the most aggressive biology and the greatest need for immunotherapy—are disproportionately less likely to receive dinutuximab-based regimens if they are Black or Hispanic, representing a direct relationship between molecular risk and structural disadvantage [15]. The intersection of molecular risk and structural disadvantage across select PST is illustrated in Figure 2.

On the global scale, these differences become even more pronounced. In low- and middle-income countries, where limited availability of advanced diagnostics, risk-adapted therapy, and supportive care infrastructure produces markedly lower survival rates, with five-year survival for high-risk neuroblastoma falling below 20% in these settings compared to rates exceeding 50% in high-income regions [14]. Additional challenges, including treatment abandonment, absence of stem cell transplant programs, and inconsistent access to essential medications, widen this gap. These patterns point to the need for clinical approaches that prioritize equitable care delivery, expand access to molecular diagnostics, and support diverse patient populations through culturally and linguistically appropriate services, while coordinated public health strategies and international collaboration remain essential to reducing disparities and improving survival across all populations.

**Figure 3:**
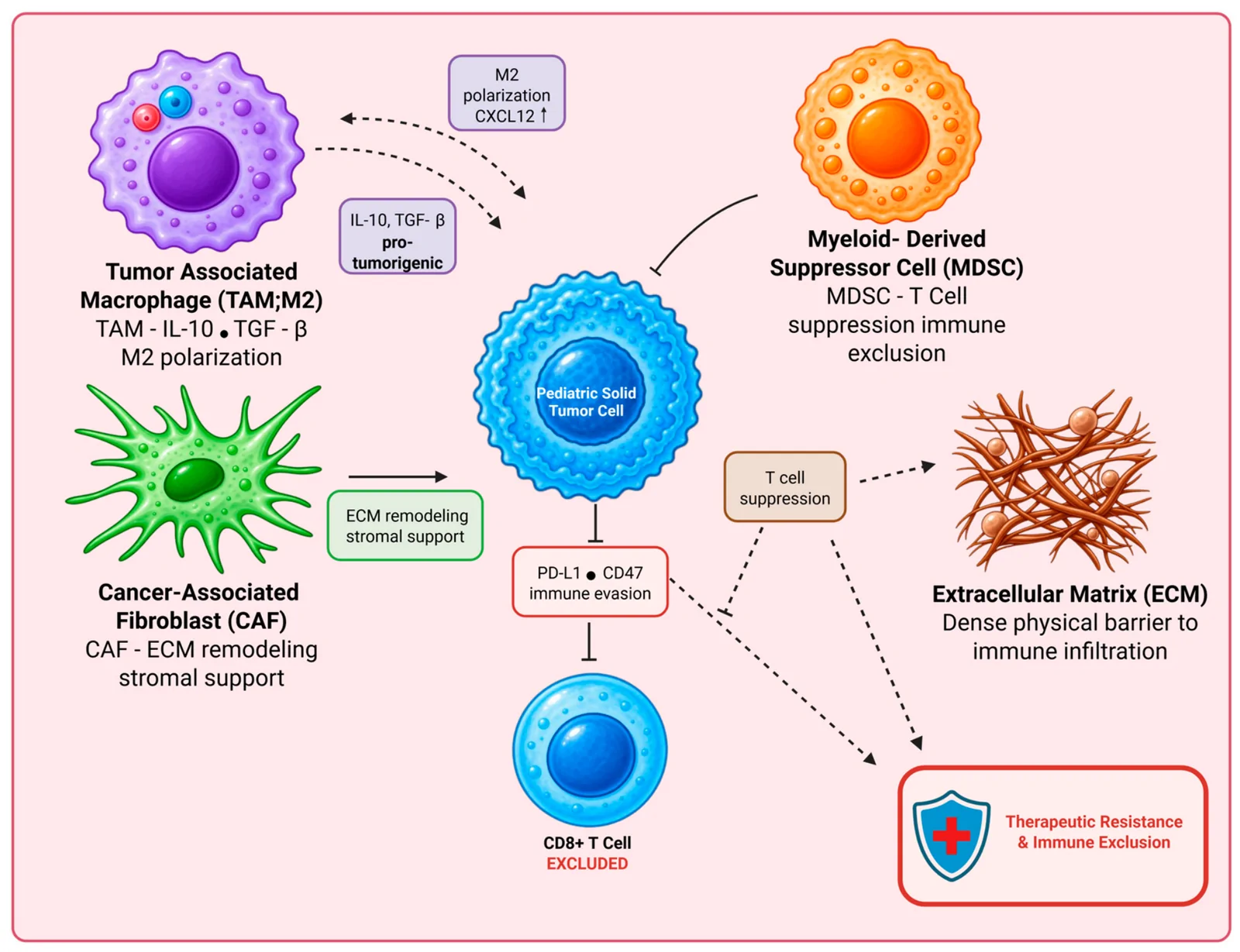
**Shared immunosuppressive tumor microenvironment architecture across select pediatric solid tumors**. Tumor-associated macrophages (TAMs), myeloid-derived suppressor cells (MDSCs), cancer-associated fibroblasts (CAFs), and extracellular matrix (ECM) establish barriers to cytotoxic T cell infiltration across tumor types, contributing to therapeutic resistance. TAMs represent a shared immunosuppressive feature documented across all six tumor types reviewed. TAM, tumor-associated macrophage; MDSC, myeloid-derived suppressor cell; CAF, cancer-associated fibroblast; ECM, extracellular matrix; PD-L1, programmed death-ligand 1; CD47, cluster of differentiation 47; **↑,** increased expression or activity, including increased CXCL12 associated with M2 macrophage polarization. Created in BioRender. Veloz, A. (2026) https://BioRender.com/5t87bht.

In parallel with these systemic influences, tumor-intrinsic biology and the local cellular context play a central role in disease progression and resistance to therapy, as this environment comprises a dynamic network of cancer-associated fibroblasts, tumor-associated macrophages, myeloid-derived suppressor cells, extracellular matrix components, and soluble signaling mediators that together establish an immunosuppressive setting [35,36]. Within this context, cancer-associated fibroblasts have been linked to enhanced invasive behavior, while tumor-associated macrophages and myeloid-derived suppressor cells impair T-cell infiltration and antitumor immune responses [37], and exposure to chemotherapy can also reshape these interactions by altering cytokine and chemokine signaling within the tumor niche, promoting the expansion of macrophage populations associated with disease recurrence [38]. The shared immunosuppressive tumor microenvironment architecture across select PST is illustrated in Figure 3.

Therapeutic strategies for neuroblastoma are determined by a risk classification that captures the marked biological and clinical variability of the disease. Patients with low- and intermediate-risk disease often achieve favorable outcomes with surgical resection alone or in combination with limited chemotherapy, whereas approximately 40–50% of patients present with high-risk disease that accounts for the majority of neuroblastoma-related mortality and requires intensive multimodal treatment.

This approach includes induction chemotherapy, surgical resection, myeloablative therapy with autologous stem cell rescue, radiotherapy, and immunotherapy targeting the disialoganglioside GD2, yet despite the use of aggressive, standardized protocols, outcomes for high-risk disease remain suboptimal. Advances in precision oncology have introduced targeted therapies, including anaplastic lymphoma kinase inhibitors, alongside immunotherapeutic approaches aimed at modulating the tumor microenvironment [32]; however, durable responses remain limited due to biological variability, resistance mechanisms, and intricate tumor–host interactions.

Neuroblastoma is a molecularly heterogeneous cancer whose outcomes are shaped by both tumor biology and sociodemographic context. Continued efforts to characterize its disease mechanisms and tumor microenvironment will be essential for developing more effective, individualized treatment strategies.

## 3. Brain Tumors

Pediatric brain tumors constitute the most common solid malignancies in children and remain the leading cause of cancer-related mortality in this population [39]. Rather than arising from the gradual accumulation of somatic mutations, these tumors are frequently linked to disruptions in early developmental programs governed by genetic and epigenetic regulators of neurodevelopment [39]. They originate from immature central nervous system lineages that are governed by developmental programs distinct from those of mature neural tissue, giving rise to biologically distinct entities with markedly different clinical trajectories and therapeutic responses.

Among the most prevalent entities are low-grade gliomas, high-grade gliomas, medulloblastomas, ependymomas, and atypical teratoid/rhabdoid tumors. Advances in molecular diagnostics, together with the 2021 World Health Organization Classification of Tumors of the Central Nervous System, have established an integrated framework that combines histopathological features with molecular characteristics, including *BRAF* alterations, histone 3 lysine 27 methylation patterns, and chromosomal changes such as 1q gain or 6q loss [40]. This framework enables refined risk stratification and directly informs clinical decision-making, particularly in medulloblastoma, which is now categorized into molecular subgroups, including *WNT*, *SHH*, Group 3, and Group 4, each associated with distinct biological behavior and clinical outcomes [40]. Clinical manifestations are determined by tumor location, size, and growth dynamics, with patients presenting with persistent headaches, nausea, vomiting, visual disturbances, motor deficits, or seizures. Evaluation typically relies on contrast-enhanced MRI for detailed anatomical and functional assessment, followed by tissue acquisition through biopsy or surgical resection to establish histological and molecular characterization [40]. Biomarker discovery efforts, together with large-scale initiatives such as the Open Pediatric Brain Tumor Atlas, have enabled extensive molecular profiling and facilitated the identification of novel therapeutic targets [16].

Structural inequities in care delivery translate directly into survival gaps, with children from historically marginalized racial and ethnic groups consistently experiencing worse outcomes than non-Hispanic White populations [16]. These differences are attributable to systemic barriers that limit access to specialized neuro-oncology services, delay diagnostic imaging and surgical referral, and complicate care delivery through language discordance, insurance limitations, and reduced health literacy [16]. Socioeconomic conditions also shape treatment access and adherence, as children from low-income households are more likely to experience delays in diagnosis, miss follow-up appointments, or discontinue therapy due to financial or logistical constraints. Insurance status has also been associated with differences in surgical management and follow-up intensity, both of which are critical for optimizing outcomes in tumors such as medulloblastoma and ependymoma [16]. Geographic factors add difficulty, particularly in rural and underserved regions where access to pediatric neurosurgical expertise and radiation oncology facilities is limited, often requiring extended travel to tertiary care centers, a burden that disproportionately affects families in states such as Montana, Wyoming, and Alaska in the United States, as well as rural populations in Latin America and sub-Saharan Africa where referral infrastructure is limited or absent [13,16].

Clinical trial participation is additionally still uneven, with children from racial and ethnic minority populations underrepresented in pediatric brain tumor studies. This imbalance limits the generalizability of findings and restricts equitable access to emerging therapies and advances in supportive care [16]. Limited representation across diverse genetic and environmental contexts also constrains the development of precision medicine approaches that are broadly applicable across populations.

At a global scale, survival differences are even more pronounced. In low- and middle-income countries, five-year survival rates for pediatric brain tumors can be reduced by 30–50% compared to those in high-income regions due to late presentation, diagnostic limitations, treatment abandonment, and inadequate supportive care infrastructure [13]. Tumors requiring intensive multimodal management and long-term follow-up, including high-grade gliomas and atypical teratoid/rhabdoid tumors, are particularly difficult to manage in these settings. Within high-income countries, persistent disparities are also observed among Indigenous populations and recent immigrant groups who may encounter additional cultural or systemic barriers to care. Efforts to address these differences require coordinated strategies that extend beyond clinical management, including expanding telemedicine for remote evaluation, developing regional pediatric oncology centers, improving language-concordant care, and implementing policies that ensure equitable insurance coverage. Inclusion of underrepresented populations in genomic research and clinical trials is essential for advancing equitable precision oncology.

Biological features within the tumor and the immune environment in which they develop, exert a major influence on disease behavior and therapeutic response. Pediatric brain tumors display a spectrum of immunological states rather than a uniform profile. High-grade gliomas frequently establish a myeloid-dominant environment through tumor-derived chemokines and hypoxia-associated signaling that promote macrophage and microglial recruitment, whereas medulloblastomas more commonly exhibit a poorly immunogenic setting characterized by limited cytokine activity and reduced lymphocyte infiltration [41,42]. Systemic immune alterations are also evident at diagnosis, including reduced lymphopoiesis and dysregulated immune activation prior to treatment initiation [43]. Tumor-derived factors and glucocorticoid-mediated signaling can also disrupt bone marrow homeostasis, leading to T-cell sequestration and peripheral lymphopenia [44]. These combined local and systemic constraints help explain the limited effectiveness of immune checkpoint blockade and adoptive cellular therapies in many pediatric brain tumors, particularly those with immune-desert or immune-excluded profiles, and support the development of approaches that address both local immunosuppression and systemic immune dysfunction [41,42].

The immune characteristics mentioned above carry important implications for therapeutic design. Immune checkpoint blockade has demonstrated limited efficacy across most pediatric brain tumors, consistent with generally low T-cell infiltration and weak baseline inflammatory signaling, although responses have been observed in molecularly defined contexts such as hypermutated or mismatch repair–deficient tumors. In pediatric high-grade gliomas, where macrophage and microglial populations are prominent, resistance appears to stem from suppressive myeloid signaling rather than absence of immune infiltration, suggesting a rationale for targeting macrophage-mediated pathways or chemokine-regulated trafficking. In medulloblastoma, where immune engagement is limited, approaches that enhance immune priming and promote intratumoral inflammation may be more appropriate, including strategies designed to recruit and activate effector cells or modify the tumor environment to support immune activity.

Adoptive cellular therapies are also under active investigation, with tumor-associated antigen targeting as a central principle. Early clinical findings indicate that CAR T cell efficacy depends on antigen selection, antigen density, intratumoral heterogeneity, and constraints imposed by the surrounding environment. Targets such as GD2 and B7-H3, along with multi-antigen or logic-gated CAR designs, are being explored to reduce antigen escape and improve tumor coverage. These observations support a treatment-matching framework in which tumors with limited immune engagement may require priming strategies prior to immune-based therapy, whereas myeloid-dominant tumors may benefit from approaches that address suppressive cellular programs and immune trafficking. Continued investigation into immune modulation and individualized therapeutic strategies is reshaping the treatment landscape.

Management of pediatric brain tumors typically involves a multimodal approach integrating surgery, radiotherapy, and chemotherapy. The extent of surgical resection is a key determinant of outcome in tumors amenable to complete removal, including ependymomas and pilocytic astrocytomas. In contrast, infiltrative or high-risk tumors arising in eloquent brain regions require additional therapeutic modalities. Radiotherapy plays a significant role in disease control but is associated with long-term neurocognitive and endocrine effects, particularly in younger patients. Chemotherapeutic regimens vary according to tumor type and molecular profile [42], and the incorporation of targeted therapies, including *BRAF* inhibitors and epigenetic modulators, into standard treatment protocols is under active investigation and shows promise in recurrent or refractory disease settings [42].

Pediatric brain tumors are a biologically heterogeneous group of malignancies whose behavior is governed by both intrinsic molecular programs and the immune context in which they develop. Outcomes have improved in certain subtypes, yet therapeutic resistance and the limited reach of immune-based strategies continue to pose central challenges, compounded by persistent inequities in access to specialized care. Progress depends on molecularly guided approaches and research frameworks that represent the full diversity of affected children.

## 4. Sarcomas

### 4.1. Ewing Sarcoma

Ewing sarcoma (ES) is one of the most frequent bone tumors of childhood and adolescence and constitutes approximately 10–15% of all bone sarcomas, with around 225 new cases diagnosed annually in individuals under 20 years of age in North America [45]. Characterized as an extremely aggressive malignancy, ES demonstrates a 5-year overall survival rate of 70–80% for patients with standard-risk, localized disease and approximately 30% for those with metastatic disease [45]. At the molecular level, ES is characterized by specific chromosomal translocations that generate fusion oncogenes encoding aberrant transcription factors. ES belongs to a broader group of pediatric sarcomas that includes osteosarcoma, rhabdomyosarcoma, and other soft tissue sarcomas [45,46]. Although considered genomically stable, rare alterations in *STAG2*, *TP53*, and *CDKN2A* may also drive aggressive phenotypes or therapeutic resistance [46,47]. The hallmark translocation t(11;22) (q24;q12), present in approximately 85% of cases, leads to the *EWS-FLI1* fusion protein, whereas alternative translocations such as t(21;22)(q22;q12), resulting in *EWS-ERG*, account for most of the remaining cases [46]. These genetic fusions reprogram the transcriptome, disrupt normal gene regulation, and promote oncogenic transformation through enhancer re-targeting and epigenetic modification [46].

The most affected anatomical sites include the pelvis, axial skeleton, and femur, although ES may arise in nearly any bone or soft tissue [45]. At diagnosis, metastases to the lungs, bone, and bone marrow are detectable in approximately 25% of patients, whereas involvement of lymph nodes, liver, or the central nervous system is rare [45]. Pain and swelling at the tumor site represent the most frequent initial symptoms, with pain often mistaken for bone growth or sports-related injury, particularly among adolescents, which leads to diagnostic delays that may range from weeks to months, with a reported median of 3–9 months [45]. On clinical examination, tendonitis is frequently suspected in adolescents, while hip inflammation or osteomyelitis may be considered in younger children [45]. In metastatic disease, patients may present with nonspecific symptoms such as malaise, fever, anorexia, and weight loss that can resemble sepsis [45].

No specific blood, serum, or urine test identifies ES; however, laboratory abnormalities may include elevated erythrocyte sedimentation rate, anemia, or leukocytosis [45]. Diagnostic evaluation typically begins with radiographs in two planes, which may show permeative osteolysis with periosteal elevation and spiculated calcifications. Magnetic resonance imaging provides the most accurate assessment of intramedullary and soft tissue extent, as well as involvement of adjacent neurovascular structures [48]. Therapeutic regimens consist of neoadjuvant multiagent chemotherapy followed by surgical resection and/or radiotherapy [45,49]. In contrast to prior reports demonstrating the success of immunotherapy in other malignancies, immune checkpoint inhibitors have shown limited efficacy in ES, with no consistent improvement in overall or progression-free survival, consistent with the immunosuppressive features within the tumor microenvironment [47]. Similarly, clinical trials evaluating intensified chemotherapy followed by autologous stem cell transplantation have failed to demonstrate reproducible survival benefit [49].

This observation contrasts with the fact that the exact cellular origin of ES is uncertain, although current evidence supports a mesenchymal stem cell that becomes locked in an undifferentiated state through *EWS-FLI1*–driven transcriptional programs. *EWS-FLI1* upregulates the histone methyltransferase *EZH2*, reinforcing epigenetic repression and maintaining tumor stemness [50]. Transcriptomic comparisons indicate that ES tumors share expression profiles with fetal, endothelial, and neural crest-derived lineages, suggesting a primitive progenitor cell at the mesenchymal neuroectodermal interface [46,50]. Supporting this concept, studies by Staege et al. demonstrated that forced expression of *EWS-FLI1* in human embryonic kidney cells failed to induce the complete ES-specific gene signature, indicating that oncogenic transformation depends not only on the fusion gene but also on the histologic context of the cell of origin [50]. The tumor microenvironment of ES is defined by an immunosuppressive cellular architecture that promotes tumor progression and limits antitumor immune responses. Tumor-associated macrophages, predominantly of the M2 phenotype, are particularly important because they promote tumor progression, immunosuppression, and angiogenesis. These macrophages may represent up to 43% of infiltrating leukocytes, suppress CD8^+^ cytotoxic T cell responses, and secrete anti-inflammatory cytokines that blunt antitumor immunity [47]. These findings support the investigation of therapeutic strategies that target macrophage depletion or reprogram macrophages toward a pro-inflammatory phenotype [47].

Disparities in ES outcomes are well documented. Although ES primarily affects non-Hispanic White adolescent males [51], incidence among Hispanic populations, including Puerto Ricans, Cubans, and South Americans, has increased over recent decades [17]. A study of Hispanic children in Florida found that older age at diagnosis, metastatic presentation, and lack of surgical resection or chemotherapy were all significantly associated with poor survival among Hispanic patients [41]. Language-based barriers, such as limited English proficiency, have also been implicated in delayed diagnosis and treatment [17]. Disparities may also point towards inequitable access to precision oncology and immunotherapy, reinforcing the need for equity-targeted interventions and increased inclusion of underrepresented populations in sarcoma research and clinical trials [17]. This is particularly consequential given that EWS-FLI1 fusion status is both the defining molecular event of ES and a marker of eligibility for emerging targeted strategies; however access to the specialized centers capable of molecular confirmation and protocol-specialized care remains unequal across racial, ethnic, and socioeconomic lines [6,17]. See Figure 3.

### 4.2. Rhabdomyosarcoma

Rhabdomyosarcoma (RMS) is the most common soft-tissue sarcoma in children, accounting for approximately 3–4% of all pediatric malignancies in the United States and nearly half of all childhood soft-tissue sarcomas [52]. The incidence is estimated at 4.71 cases per million children and adolescents aged 20 years or younger, with a modest male predominance, with a male-to-female ratio of approximately 1.3–1.5. Outcomes remain limited, with a survival rate of 63% in localized presentations and less than 20% in individuals with metastatic disease despite the use of multiagent therapeutic regimens [18]. RMS arises from primitive mesenchymal precursors committed to skeletal muscle differentiation and can develop in a wide range of anatomical sites, most frequently in the head and neck region, the genitourinary tract, and the extremities [53]. Although uncommon in adults, it is an important focus in pediatric oncology given its aggressive behavior, heterogeneous biology, and the limited therapeutic advances over the past two decades.

Histopathologic classification distinguishes several forms with unique biological and clinical profiles, most prominently the embryonal and alveolar variants, as well as less frequent subtypes such as pleomorphic, spindle, and mixed. Embryonal tumors comprise approximately 60–70% of pediatric cases and tend to arise in younger children, often involving the head, neck, and genitourinary regions. This form is associated with loss of heterozygosity at 11p15.5 and frequent alterations in RAS signaling components, including HRAS, NRAS, and KRAS, and is generally linked to more favorable outcomes, with 5-year overall survival exceeding 70%. In contrast, the alveolar form accounts for roughly 20–25% of cases and is associated with a more aggressive clinical course, typically affecting adolescents and young adults and often involving the extremities. These tumors are defined by chromosomal rearrangements that generate *PAX3–FOXO1* or *PAX7–FOXO1* fusion oncogenes through translocations such as t(2;13) or t(1;13), and are associated with reduced survival, with rates ranging from 50–60% [19,54]. Pleomorphic variants are rare in pediatric populations and may histologically resemble other sarcomas, with variable outcomes and occasional *MYOD1* mutations, whereas spindle cell forms are associated with a relatively favorable prognosis. Mixed presentations exhibit overlapping morphologic and molecular features, with clinical behavior largely determined by dominant characteristics and fusion status [54]. The 2020 World Health Organization classification emphasizes *FOXO1* rearrangement status as a central determinant of diagnosis and risk stratification [19]. A summary of the histological subtypes and their key molecular and clinical features is provided in Table 1.

Molecular characterization reveals distinct mechanisms of tumor development and clinical behavior. Alveolar disease is defined by *PAX–FOXO1* fusion oncogenes that function as aberrant transcriptional regulators, disrupting normal myogenic differentiation and promoting aggressive phenotypes. These fusion-positive tumors consistently correlate with inferior outcomes, reinforcing the importance of molecular classification beyond histologic appearance. In contrast, embryonal tumors lack recurrent fusion events and instead exhibit a diverse mutational landscape involving pathways that regulate proliferation, differentiation, and genomic stability. This variability accounts for the wide clinical spectrum observed in fusion-negative disease and increasingly influences therapeutic decision-making. Together, these distinctions illustrate the expanding role of genomic profiling in refining prognosis and guiding targeted treatment strategies.

Genomic studies have also identified a significant contribution of inherited susceptibility in pediatric RMS. A comprehensive analysis by Martin-Giacalone et al. reported that approximately 7.3% of patients carry pathogenic or likely pathogenic germline variants, particularly involving genes such as *TP53*, *NF1*, *HRAS*, *BRCA2*, and *DICER1* [19]. These variants are more frequently observed in fusion-negative tumors and are associated with established cancer predisposition syndromes, including Li-Fraumeni, Noonan, Costello, and Beckwith-Wiedemann. These observations support the incorporation of germline testing into routine clinical evaluation, even in the absence of a documented family history, and highlight the importance of integrating genomic surveillance into pediatric oncology practice.

Clinically, RMS often presents as a rapidly enlarging mass that may initially be painless. Manifestations depend on tumor location, with orbital involvement leading to proptosis or visual disturbance, head and neck disease associated with nasal obstruction or cranial nerve dysfunction, and genitourinary tumors presenting with hematuria or urinary obstruction. The botryoid variant, characterized by a polypoid mass arising from mucosal surfaces such as the bladder or vagina, is primarily observed in infants. Lesions in the extremities, more frequently associated with alveolar histology in adolescents, may resemble benign processes, leading to delays in recognition. Diagnosis is established through imaging, histopathologic evaluation, and immunohistochemical staining for muscle-specific markers, including myogenin, desmin, and MyoD1, while definitive subclassification increasingly depends on molecular techniques such as fluorescence in situ hybridization or reverse transcription PCR to detect *FOXO1* rearrangements [19].

Risk assessment integrates tumor-node-metastasis staging with surgical and clinical grouping systems to guide therapeutic selection. The development of intensive multimodal protocols through large international trials has improved survival in patients with localized disease. Even with aggressive treatment combining surgery, high-intensity chemotherapy, and radiation, outcomes remain poor in high-risk and metastatic settings. This limitation has directed attention toward tumor-intrinsic properties and the local cellular context as contributors to resistance. Although substantial progress has been made in identifying molecular drivers, the understanding of the tumor microenvironment is comparatively limited. Available evidence indicates that the immune context contributes to disease progression and reduced therapeutic responsiveness. Fusion-positive tumors frequently exhibit low lymphocyte infiltration, increased expression of immune checkpoint molecules, and suppressive cytokine activity, consistent with an immune-excluded phenotype that may facilitate immune evasion and may account for the limited benefit observed with checkpoint inhibition. Preclinical work has also highlighted roles for macrophage infiltration and *PD-L1* expression, supporting the exploration of immune-modulating strategies [56].

Disparities in disease burden and outcomes represent an important dimension of RMS epidemiology. The highest incidence occurs in early childhood, with more than half of cases diagnosed before 10 years of age, accompanied by a modest male predominance. Population-based analyses indicate higher incidence rates among non-Hispanic White children compared with non-Hispanic Black children, while Hispanic populations demonstrate intermediate rates [20]. Survival differences persist, with Black and Hispanic children experiencing worse outcomes, attributable to delays in diagnosis, limited access to specialized care, and underrepresentation in clinical trials [19]. The clinical significance of these disparities deepens when considering the molecular heterogeneity of RMS. PAX-FOXO1 fusion-positive alveolar RMS carries the worst prognosis among RMS subtypes, yet accurate fusion detection through FISH or PCR requires access to specialized molecular diagnostics that is inequitably distributed across racial, ethnic and geographic lines [19,20]. The convergence of these molecular and structural factors across neuroblastoma, Ewing Sarcoma, and rhabdomyosarcoma is summarized in Figure 3.

Additionally, data from Puerto Rico and other regions in Latin America remain limited; however, existing evidence of structural inequities in healthcare access suggests that comparable disparities may exist. Environmental and perinatal exposures have also been explored, with reported associations including advanced paternal age, parental substance use, and prenatal exposure to radiation or diagnostic imaging, although these findings remain observational and do not establish causality [52].

Therapeutic management relies on a multimodal approach incorporating chemotherapy, surgical resection, and radiation. Standard regimens typically include vincristine, actinomycin D, and cyclophosphamide. Surgical intervention is pursued when feasible, while radiation is employed for local control in cases where complete resection cannot be achieved without significant morbidity [49]. Treatment decisions are guided by classification systems developed by the Children’s Oncology Group, which incorporate tumor size, anatomical location, nodal involvement, metastatic spread, and molecular characteristics, including fusion status [19]. Despite these advances, survival for patients with advanced disease is below 30%, highlighting the need for improved strategies.

Ongoing investigations are focused on overcoming these limitations through targeted therapies and immune-based approaches. Clinical trials are evaluating MEK inhibition in tumors harboring RAS pathway alterations and adoptive cellular therapies such as CAR T cells in fusion-positive disease [20,55,56]. Translation of these approaches into meaningful clinical benefit is challenging, particularly in the context of a suppressive immune environment and limited pediatric-specific drug development pipelines. Future directions emphasize expanded access to genetic testing, integration of multi-omics approaches, and refinement of risk-adapted strategies that account for both intrinsic tumor biology and surrounding cellular interactions [20,55,56].

## 5. Wilms Tumor

Wilms tumor (WT), also known as nephroblastoma, is the most common primary renal malignancy in children, accounting for nearly 90% of pediatric kidney tumors and representing the most frequent abdominal neoplasm in this population. On a global scale, it ranks as the fourth most common malignancy among children younger than 15 years of age [58]. The disease predominantly affects early childhood, with peak incidence between three and four years, and an estimated occurrence of 1 in 10,000 live births across Europe and North America [21]. In contrast to adult renal cancers, which are typically epithelial in origin, WT arises from embryonic tissue derived from persistent metanephric blastema. This embryonic origin accounts for its rapid growth but also explains the relatively favorable response to therapy seen in most cases [59]. Clinically, WT often presents as a large, firm, and painless abdominal mass that is frequently identified incidentally. Although many patients remain asymptomatic, additional findings may include hematuria, hypertension, abdominal discomfort, fever, or reduced appetite [60]. Radiologic evaluation is essential for establishing diagnosis, defining disease extent, and guiding treatment planning.

On histopathologic evaluation, Wilms tumor displays a triphasic composition in which the relative proportion of each component varies across cases and carries established prognostic significance. Classification into favorable and unfavorable histology remains central to clinical management, with diffuse anaplasia representing the principal unfavorable subtype and strongly correlating with *TP53* alterations and reduced survival [61]. Congenital syndromes involving genitourinary anomalies, overgrowth, or WT1 dysregulation, exemplified by Beckwith-Wiedemann and Denys-Drash syndromes, account for approximately 10% of cases and underscore the importance of genetic counseling and longitudinal imaging surveillance in high-risk populations [62]. Recognition of these syndromic associations highlights the importance of genetic counseling and longitudinal imaging surveillance in high-risk populations.

The molecular landscape of WT involves a combination of genetic alterations and epigenetic dysregulation that influence tumor initiation and progression. A well-established pathogenic mechanism involves mutations or deletions affecting the *WT1* gene located on chromosome 11p13, a tumor suppressor with critical roles in renal and gonadal development. WT1 expression is observed in the metanephric blastema as well as in the gonadal ridge, spleen, and mesothelial tissues. Alterations affecting the 11p15 region, including loss of imprinting or uniparental disomy involving the IGF2 and H19 loci, represent another major driver, particularly in association with Beckwith–Wiedemann syndrome [63]. Although the designation “*WT2*” is sometimes used to refer to this locus, it does not correspond to a single defined gene but rather encompasses this epigenetically regulated region [64]. Additional recurrent alterations include activating mutations in *CTNNB1*, amplification of MYCN, inactivation of *WTX*, and changes affecting *TP53*, each contributing to dysregulated proliferation, impaired differentiation, or resistance to therapy [65]. Additional details regarding these genetic features are summarized in Table 2.

Despite this molecular heterogeneity, WT is associated with one of the most favorable survival outcomes among pediatric solid tumors, with five-year overall survival exceeding 90% in high-resource settings [21]. These outcomes are the product of coordinated efforts of international cooperative groups, including the Children’s Oncology Group and the Société Internationale d’Oncologie Pédiatrique, which have established complementary treatment strategies. In the United States, management commonly begins with primary nephrectomy followed by stage- and histology-directed chemotherapy and, when indicated, radiotherapy. European protocols opt to frequently incorporate preoperative chemotherapy to reduce tumor burden and minimize surgical complications, particularly intraoperative rupture, which is a significant adverse prognostic factor [66]. Treatment planning incorporates tumor stage, histological classification, specific genomic alterations such as loss of heterozygosity at chromosomes 1p and 16q, and response to initial therapy. Standard management includes nephrectomy combined with vincristine- and actinomycin-based regimens, with the addition of doxorubicin for higher-risk disease, and selective use of radiation in advanced-stage or high-risk cases. Although these approaches have improved survival, long-term sequelae remain a concern, particularly in patients exposed to anthracyclines or abdominal irradiation, which may result in cardiotoxicity, growth abnormalities, renal impairment, and secondary malignancies [21].

Marked differences in outcomes persist across regions and populations. In low- and middle-income countries, survival is significantly reduced due to delayed diagnosis, limited access to imaging and treatment, shortages of essential medications, and interruptions in care delivery [11]. Reports from sub-Saharan Africa indicate five-year survival rates below 50%, with treatment abandonment attributable to financial and logistical constraints [12]. Within the United States, disparities are also evident, as Black and Hispanic children are more likely to present with advanced disease and may experience barriers to optimal care despite evidence suggesting comparable tumor biology [67]. These patterns call for coordinated efforts that address both structural limitations and access to care, including investment in healthcare infrastructure, improved education, and culturally responsive patient support systems.

Research efforts continue to refine therapeutic approaches through improved risk stratification and integration of molecular data. Advances in next-generation sequencing and methylation profiling are enhancing the classification of WT subtypes and enabling identification of markers associated with relapse. Circulating tumor DNA is being explored as a potential tool for monitoring minimal residual disease and assessing treatment response [68]. Aberrant activation of signaling pathways, including IGF2 and Wnt/β-catenin, has been consistently identified, supporting their relevance as both prognostic indicators and potential therapeutic targets. Translation of these findings into clinical practice is ongoing, highlighting the need for translational studies that connect molecular findings to effective clinical intervention.

## 6. Retinoblastoma

Retinoblastoma (RB) is an uncommon but clinically important pediatric malignancy that accounts for approximately 2–3% of childhood cancers and represents the most common intraocular tumor in infancy and early childhood. It originates from retinal precursor cells during ocular development and is most frequently diagnosed before five years of age, with a global incidence ranging from 1 in 15,000 to 20,000 live births [69]. RB has been central to the understanding of cancer genetics, serving as the model through which Alfred Knudson proposed the “two-hit hypothesis,” describing how biallelic inactivation of tumor suppressor genes can initiate tumorigenesis [70]. Disease classification distinguishes heritable from non-heritable forms based on the presence of germline mutations in the *RB1* gene located on chromosome 13q14. Approximately 40% of cases are heritable and commonly present as bilateral and multifocal tumors, whereas the remaining 60% are typically unilateral and non-familial [71,72]. Despite this distinction, a subset of children with unilateral disease, estimated at 10–15%, harbor germline *RB1* alterations and therefore require genetic evaluation and long-term monitoring [72]. The RB1 gene encodes a nuclear phosphoprotein that regulates the transition from the G1 to S phase of the cell cycle, and tumor formation occurs when both alleles are inactivated either through inherited mutation followed by somatic loss or through two independent somatic events [72,73,74]. A smaller subset of tumors lacks RB1 alterations and is instead defined by amplification of the MYCN oncogene. These cases tend to be unilateral, non-heritable, and present at a younger age, often within the first six months of life, with features of high proliferative capacity, poor differentiation, and more aggressive clinical behavior, highlighting the importance of molecular testing in early-onset unilateral disease [74].

Clinical presentation most commonly includes leukocoria and strabismus, although additional findings may involve decreased vision, orbital swelling, or proptosis in more advanced stages [71]. Evaluation typically involves examination under anesthesia with fundoscopy, supported by ocular ultrasound and magnetic resonance imaging of the brain and orbits to assess optic nerve involvement and intracranial extension. In selected cases where extraocular spread is suspected, lumbar puncture or bone marrow biopsy may be required for staging [75]. Histologically, RB consists of densely packed basophilic cells forming characteristic structures such as Flexner–Wintersteiner or Homer Wright rosettes, with varying degrees of necrosis and calcification. Certain pathological features, including massive choroidal invasion, post-laminar optic nerve involvement, and anterior chamber extension, are associated with increased risk of systemic dissemination and are most reliably identified following enucleation [73]. Detection of these features guides the need for adjuvant chemotherapy even in the absence of clinically evident metastasis.

Therapeutic strategies have evolved substantially over recent decades, with increasing emphasis on globe preservation when feasible. Enucleation is necessary for advanced tumors without visual potential or with extraocular extension. Systemic chemotherapy, most commonly combining vincristine, etoposide, and carboplatin, continues to play a central role, particularly in bilateral or multifocal disease [76]. Targeted delivery approaches have transformed management in localized settings. Intra-arterial chemotherapy, involving direct administration of melphalan through the ophthalmic artery, has demonstrated high effectiveness in advanced unilateral disease and enables eye preservation in a substantial proportion of patients while minimizing systemic exposure [77]. Intravitreal chemotherapy has also become standard for the management of vitreous seeding, which historically represented a major indication for enucleation. Agents such as melphalan or topotecan achieve high intraocular concentrations and provide effective disease control with limited toxicity [78]. Local therapies, including transpupillary thermotherapy, laser photocoagulation, and cryotherapy, remain important for smaller lesions or as adjuncts to systemic or intra-arterial approaches. Treatment selection is guided by disease classification, laterality, tumor location, and anticipated visual outcomes. Systems such as the International Intraocular Retinoblastoma Classification and the Reese–Ellsworth scale are widely used to standardize management, with earlier-stage disease often managed conservatively and more advanced stages requiring aggressive intervention.

Survival outcomes differ markedly across regions. In high-income countries, survival exceeds 95% for intraocular disease, whereas outcomes remain poor in low- and middle-income settings, where more than half of patients present with extraocular or metastatic disease as a consequence of delayed diagnosis and limited access to specialized care [22]. Studies from Central and South America, sub-Saharan Africa, and South Asia report mortality rates exceeding 50%, attributable by advanced presentation, diagnostic limitations, treatment interruption, and financial barriers. These challenges have led the World Health Organization to designate RB as a priority within the Global Initiative for Childhood Cancer, promoting early detection, referral networks, and access to multidisciplinary care [23]. Disparities are also observed within high-resource environments. In the United States, children from underrepresented racial and ethnic groups are more likely to present with advanced disease, undergo enucleation, and are less likely to receive genetic testing or intra-arterial therapy compared with non-Hispanic White patients, emphasizing inequities in access to subspecialty care and referral pathways [24]. The biology of RB, driven by biallelic RB1 inactivation and highly responsive to early intervention, makes delayed diagnosis in LMIC settings a structural failure with preventable biological consequences, as tumors that would be treatable with globe-preserving approaches in high-income settings progress to extraocular disease requiring enucleation or causing death [22,23].

Long-term management is particularly important for individuals with heritable diseases, as the lifetime risk of secondary malignancies is elevated. Osteosarcoma, soft tissue sarcoma, and melanoma are among the most frequently observed secondary cancers, especially in patients previously exposed to radiation therapy [79]. Survivorship care, therefore, includes ongoing surveillance for secondary neoplasms, prosthetic management when applicable, psychosocial support, and regular ophthalmologic evaluation. Family members may also require genetic counseling and screening based on inheritance patterns.

Current research efforts aim to refine diagnostic approaches and expand therapeutic options. Liquid biopsy approaches interrogating aqueous humor and plasma-derived nucleic acids are emerging as minimally invasive tools for molecular tumor characterization and recurrence monitoring [80]. Parallel investigational efforts are targeting RB1-deficient cells through synthetic lethality, while immune-based and gene-editing approaches are being developed to expand the therapeutic landscape in refractory disease. Incorporation of molecular profiling into routine clinical practice has the potential to enable more precise risk-adapted treatment and improve outcomes in this disease [72,74].

The retinoblastoma tumor microenvironment has emerged as an area of increasing scientific interest. Single-cell transcriptomic analyses have revealed that RB tumors harbor a heterogeneous cellular ecosystem comprising malignant cone progenitor cells in varying states of differentiation, tumor-associated macrophages, endothelial cells, and cancer-associated fibroblasts, with distinct cell populations displaying differential expression of immune checkpoint molecules including PD-L1 and CD47 [81]. Macrophage infiltration has been identified as a prominent feature of the RB microenvironment, and the polarization state of these cells appears to correlate with tumor aggressiveness and treatment response. These findings provide a cellular and molecular rationale for exploring immune-based strategies in RB, particularly in high-risk or refractory disease where conventional approaches have failed. The translation of these insights into clinical applications, including the targeting of immune checkpoints or the reprogramming of myeloid cells, represents a compelling direction for future research.

## 7. Malignant Bone Tumors

### 7.1. Osteosarcoma

Pediatric osteosarcoma is an aggressive malignancy known as the most common primary bone cancer in children and adolescents. It accounts for approximately 2–3% of childhood cancers worldwide and nearly 20% of primary bone tumors in this age group. The estimated global incidence ranges from 3 to 5 cases per million individuals younger than 20 years, with a peak occurring during the adolescent growth spurt between 10 and 19 years of age [82]. During this phase of accelerated skeletal development, tumors most frequently arise in the metaphyseal regions of long bones, particularly the distal femur, proximal tibia, and proximal humerus, areas characterized by intense osteogenic activity.

Clinical presentation is typically marked by progressively worsening localized pain, swelling, and functional limitation. Early manifestations are frequently misattributed to minor trauma or growth-related discomfort, leading to delays in diagnosis that allow disease progression. As tumor burden increases, patients may develop visible deformities or sustain pathological fractures. At the time of diagnosis, approximately 15–20% of patients have radiographically evident pulmonary metastases, while a larger proportion are presumed to harbor subclinical metastatic disease [48].

Radiologic evaluation is essential for both diagnosis and staging. Plain radiographs commonly demonstrate aggressive lesions with mixed lytic and sclerotic features, cortical disruption, and periosteal reactions such as Codman triangle formation or a sunburst pattern. Magnetic resonance imaging provides detailed assessment of intramedullary involvement and extension into surrounding soft tissues and neurovascular structures, information that is critical for surgical planning. Computed tomography of the chest is required for detection of pulmonary metastases, whereas bone scintigraphy or positron emission tomography supports evaluation of skeletal spread. Definitive diagnosis relies on histopathologic confirmation through core needle or open biopsy, allowing accurate subtype classification and exclusion of conditions such as Ewing sarcoma or infectious processes [82].

Marked histologic diversity characterizes osteosarcoma. The osteoblastic form is most frequently encountered, although chondroblastic, fibroblastic, telangiectatic, small cell, and epithelioid variants are also recognized. Surface lesions, including parosteal and periosteal types, generally display lower-grade features and slower growth kinetics [82]. In contrast, high-grade central or conventional osteosarcoma represents approximately 80–90% of pediatric cases and constitutes the dominant clinical entity, accounting for the majority of morbidity and mortality [48,82].

At the molecular level, osteosarcoma is characterized by extensive chromosomal instability, copy number alterations, and intricate structural rearrangements. Multi-omics approaches have identified biologically distinct subgroups with differing clinical implications, including tumors with immune-enriched profiles, those with minimal immune infiltration, cases dominated by homologous recombination deficiency, and tumors with MYC-related pathway activation, each associated with unique transcriptional signatures and clinical behavior [83]. Differences between pediatric and adult disease are also evident, with higher frequencies of *TP53*, *FLCN*, and *NCOR1* alterations observed in younger patients, suggesting that age-specific molecular targeting strategies may be required [84].

The tumor microenvironment has emerged as a critical determinant of disease behavior, influencing progression, metastatic dissemination, and response to therapy. It is defined by dynamic interactions among immune cells, stromal elements, and vascular components, coordinated through cytokine and chemokine signaling networks that shape both tumor growth and local immune suppression. Gene expression analyses at diagnosis indicate that variation in immune-related signatures can distinguish subgroups with different clinical outcomes [85]. Complementary immunogenomic studies demonstrate that some tumors exhibit markedly reduced immune cell infiltration, a feature that may add to the limited effectiveness of immune checkpoint blockade in this disease [86]. These observations highlight the clinical importance of microenvironmental diversity and support efforts aimed at modifying local immune conditions as part of therapeutic strategy.

Outcome differences remain closely linked to broader structural factors. Children from underrepresented racial and ethnic backgrounds, including Black and Hispanic populations, encounter barriers to early diagnosis, access to specialized treatment centers, and participation in clinical research [8,9,87]. These factors are associated with more advanced disease at presentation and reduced survival, even in settings with otherwise developed healthcare systems [87]. Socioeconomic context also shapes clinical outcomes. Patients from lower-income households often experience delays in diagnostic evaluation and face logistical challenges that interfere with adherence to treatment protocols. Lower parental education levels and instability in insurance coverage, including reliance on public insurance or lack of coverage, have been associated with decreased likelihood of receiving limb-sparing procedures, delays in chemotherapy initiation, and reduced access to high-volume oncology centers [25]. Geographic factors also play a role, as individuals residing in rural or underserved regions may encounter limited access to specialized care, increased travel burden, and fragmented follow-up. Data from Eastern Europe indicate that rural patients present with larger tumors, demonstrate lower treatment adherence, and experience poorer survival compared with urban populations [26]. Within the United States, analyses from national registries such as SEER and institutional cohorts consistently show that Black and Hispanic children have lower five-year survival rates than non-Hispanic White patients, even after accounting for stage and treatment differences [25]. Language barriers, implicit bias, and limited inclusion in clinical trials likely contribute to these disparities. Reduced participation of Hispanic patients in molecular studies and innovative trials restricts access to precision-based therapies.

On a global scale, survival is substantially lower in low- and middle-income countries, where reported outcomes frequently fall below 20%. Key factors include delayed diagnosis, limited availability of multimodal treatment, shortages of specialized surgical and oncology services, and high rates of treatment abandonment [11,12]. These patterns emphasize the need for system-level interventions, including policy-driven equity initiatives, culturally responsive care models, and international collaborations aimed at strengthening pediatric oncology infrastructure in resource-limited settings [13].

Management of pediatric osteosarcoma relies on a multimodal approach that combines neoadjuvant chemotherapy, definitive surgical resection, and postoperative chemotherapy. Limb-salvage procedures are preferred when feasible. The MAP regimen, consisting of high-dose methotrexate, doxorubicin, and cisplatin, is the backbone of systemic therapy [27,88]. In high-income settings, five-year overall survival for localized pediatric osteosarcoma is approximately 70%, whereas metastatic disease carries a five-year survival of approximately 20–30% [27]. Outcomes for patients with metastatic or recurrent disease remain poor, and progress has been limited over the past two decades, prompting increased interest in novel therapeutic strategies.

Current research efforts focus on integrating targeted and immune-based approaches. Investigational strategies include the use of poly (ADP-ribose) polymerase inhibitors in tumors with homologous recombination defects and kinase inhibitors targeting dysregulated signaling pathways [83,84]. In parallel, immune-directed approaches such as macrophage reprogramming, T-cell–engaging therapies, and tumor-specific vaccine platforms are being explored to overcome resistance associated with suppressive local immune conditions [85,86]. The 2023 European Standard Clinical Practice Guidelines highlight the importance of incorporating molecular diagnostics, biologically informed risk stratification, and collaborative international trials to address the persistent lack of therapeutic advancement in pediatric osteosarcoma [89].

Pediatric osteosarcoma is a genomically complex disease whose management requires integration of molecular insight with equitable access to care. Continued investigation into underlying mechanisms, development of innovative treatment strategies, and focused efforts to address systemic disparities remain essential for improving both survival and long-term outcomes in affected patients [45,83,87].

### 7.2. Chondrosarcoma

Chondrosarcoma is a malignant cartilage-forming tumor that is exceedingly uncommon in pediatric populations, accounting for well under 1% of childhood malignancies and representing a minor proportion of pediatric bone tumors [90,91]. In contrast to adult presentations, pediatric cases often exhibit differences in clinical behavior and disease course, creating challenges in both recognition and management [90]. The pelvis, proximal femur, and humerus are among the most frequently involved sites, with patients typically presenting with gradually worsening pain, localized swelling, and decreased joint mobility [90,91]. Evaluation relies heavily on imaging, with magnetic resonance imaging and computed tomography used to define tumor extent and guide operative planning [92]. Conventional radiographs may demonstrate characteristic chondroid mineralization patterns, including rings-and-arcs calcifications. Tissue sampling is necessary to establish tumor grade and subtype. In pediatric cases, mesenchymal chondrosarcoma is more common than in adult populations and is a particularly aggressive form. This variant is characterized by recurrent gene fusions, most notably *HEY1–NCOA2*, and is associated with early dissemination and an unfavorable clinical course [91,93].

Genetic and epigenetic alterations drive disease development and progression. Recurrent mutations have been identified in *IDH1* and *IDH2*, *TP53*, and *EXT1/2*, along with dysregulation of signaling pathways including PI3K–AKT, IHH–GLI1, and c-MYC. Epigenetic changes, such as DNA hypermethylation and increased expression of regulators like *SIRT1*, also influence tumor behavior and resistance to therapy [93,94]. These molecular findings portray the biological heterogeneity of chondrosarcoma and support ongoing efforts to identify actionable targets for individualized treatment.

The local cellular environment represents a major obstacle to therapeutic intervention. Chondrosarcomas are typically characterized by low vascularity, dense calcified matrix, and a rigid extracellular framework that limits immune cell infiltration. These structural constraints, combined with dysregulated checkpoint signaling and reduced lymphocyte presence, contribute to limited responsiveness to immune-directed therapies [95,96,97]. Addressing these barriers will likely require strategies that modify the local tumor environment to improve immune accessibility and enhance treatment response.

Outcome differences are strongly influenced by structural and socioeconomic factors. Children from disadvantaged backgrounds are more likely to encounter delays in diagnosis, reduced access to specialized sarcoma centers, and lower participation in clinical research, patterns observed across multiple rare pediatric malignancies [7,8,9,87]. Racial and ethnic inequities also affect care delivery. In the United States, Black and Hispanic children are more likely to present with advanced disease and experience barriers to subspecialty care, even when treatment resources are available [87]. Contributing factors include insurance instability, language barriers, geographic limitations, and reduced access to culturally responsive care, all of which impact outcomes across the continuum of pediatric oncology [7,87]. Advancement in this field depends on deeper molecular characterization and improved access to targeted therapies. Precision approaches, including *IDH* inhibition and modulation of PI3K–mTOR signaling, have shown potential in selected patient subsets [95,96,97]. Expanding access to clinical trials and addressing structural limitations in healthcare delivery remain essential to improving outcomes for children affected by this rare malignancy.

From a clinical management perspective, chondrosarcoma is notable for its intrinsic resistance to conventional chemotherapy and radiotherapy, a characteristic that distinguishes it from most other pediatric sarcomas and underscores the role of surgical resection in achieving curative intent. Complete resection with wide negative margins represents the cornerstone of primary management. Thus, it remains among the strongest independent predictors of local recurrence and disease-specific survival. In pediatric patients, achieving adequate margins often requires demanding reconstructive procedures and carries implications for long-term functional outcomes, including limb-length discrepancy, joint dysfunction, and prosthetic management. For mesenchymal chondrosarcoma, where systemic dissemination occurs early and the risk of relapse is high, multiagent chemotherapy is incorporated into treatment protocols in a manner analogous to other high-risk pediatric sarcomas, although evidence from pediatric-specific cohorts is limited. Emerging systemic approaches, including targeted inhibition of IDH1 and IDH2 and modulation of the hedgehog signaling axis, are under active preclinical and early clinical evaluation, with the goal of overcoming chemoresistance and expanding treatment options for patients with unresectable or metastatic disease [95,96,97].

Chondrosarcoma in the pediatric population represents a rare and clinically challenging entity whose rarity has historically limited the development of dedicated treatment protocols and evidence-based guidelines. The marked biological heterogeneity across subtypes, from low-grade conventional lesions with indolent behavior to the highly aggressive mesenchymal variant, demands individualized clinical evaluation and multidisciplinary expertise. Continued molecular characterization, integration of pediatric patients into collaborative international trials, and investment in translational models that recapitulate the unique biology of pediatric chondrosarcoma will be essential for advancing outcomes in this underserved population.

## 8. Conclusions

The eight tumor types examined in this review collectively demonstrate that pediatric solid tumors cannot be understood through biology alone. Across the selected PSTs, molecular characterization has transformed risk stratification and informed therapeutic design; however, survival gaps persist and, in many cases, have widened along socio-economic lines and demographic backgrounds.

The molecular evidence is equally prominent. MYCN amplification identifies the highest-risk neuroblastoma subgroup, but evidence shows Black and Hispanic children with MYCN-amplified disease are less likely to receive dinutuximab—the immunotherapy regimen their molecular profile demands [15]. PAX-FOXO1 fusion status is the single strongest predictor of poor outcomes in rhabdomyosarcoma, rendering FISH and PCR assays crucial for detection but still unequally distributed across racial and geographic lines [19,20]. EWS-FLI1 fusion drives the transcriptional reprogramming central to Ewing sarcoma biology, yet the specialized centers capable of molecular confirmation and protocol-driven management are also not equally reachable by all patients [6,17]. Across all tumor types reviewed, the tumor microenvironment functions as a shared mediator of immune exclusion dominated by tumor-associated macrophages and suppressive myeloid populations. Although the latter has been confirmed, immune-based strategies have demonstrated consistent clinical benefit only in neuroblastoma, underscoring both the promise and current limitations of this therapeutic approach. These are not isolated observations, but a pattern, illustrated in Figure 3.

What this pattern clearly reveals is that the science of pediatric solid tumors and the structural conditions of pediatric cancer care are not separate problems. A child who carries MYCN amplification and lives in a resource-limited setting faces a compounding biological and structural disadvantage that neither precision oncology nor healthcare policy can resolve independently. Advancing this field requires trials that enroll the populations most affected, genomic databases that include ancestry-derived samples, and care delivery systems that close the distance between a molecular finding and the therapy it justifies. With biological advancements must come an infrastructure that parallels its pace.

The evidence synthesized in the review is not abstract; biological risk and structural disadvantage co-occur in pediatric oncology in ways that are not incidental. Pediatric patients who carry the most aggressive molecular subtypes are disproportionately those with the least access to the diagnostics and therapies those subtypes require. Addressing such requirements necessitates not only continued investment in translational science, but a deliberate expansion of what counts as scientific rigor, including population diversity in the fields expressed above. The standard for progress in pediatric oncology must be defined by who benefits, not only by what is discovered.

This review has several limitations that should be acknowledged. As a narrative review, it does not follow a systematic or meta-analytic methodology, and the selection of literature, while guided by established search criteria, may not capture all relevant studies. The scope was intentionally limited to eight major pediatric solid tumor types, and several clinically important entities, including hepatoblastoma, germ cell tumors, and other rare malignancies, were not included. The evidence base for health disparities in pediatric oncology, while growing, is uneven across tumor types and geographic regions, and findings from high-income settings may not be generalizable to low- and middle-income countries. Comparative survival data across international settings were not systematically compiled, and the integration of Latin American, Asian, and sub-Saharan African data is still limited by the availability of published population-based studies in these regions. Additionally, the rapidly evolving nature of the field means that some developments published close to or after the literature search window may not be captured in this review.

## Figures and Tables

**Table 1:** Histological and molecular subtype divisions of pediatric rhabdomyosarcoma (RMS). Survival estimates are based on general outcomes reported in the literature and vary by stage, age, and treatment protocol.

Histological Subtype	Description
Embryonal	Most common occurring subtype (60–70% of pediatric RMS). Typically affects children under 10 years of age and arises in the head, neck, and genitourinary tract. Characterized by loss of heterozygosity at chromosome 11p15.5, RAS pathway mutations (e.g., *HRAS*, *NRAS*, *KRAS*), and better overall prognosis with 5-year overall survival > 70%. Fusion-negative [20,55,56].
Alveolar	Less common (20–25%) but more aggressive. Affects adolescents and young adults. Defined by *PAX3/7-FOX01* fusion oncogenes via t (2;13) or t (1;13). Associated with higher metastatic potential and lower 5-year overall survival (50–60%) [19,54].
Pleomorphic	Rare variant. May resemble other sarcomas histologically. The prognosis is variable; some pediatric cases have an indolent course. Often fusion-negative but harbor *MYOD1* mutations [19].
Spindle	Rare subtype accounting for up to 10% of pediatric RMS cases. More common in the paratesticular region and head and neck in pediatric patients. Molecularly heterogeneous: infantile cases harbor VGLL2/NCOA2 gene fusions and carry favorable prognosis; tumors in older children with MYOD1 mutations follow an aggressive clinical course with poor outcomes. Molecular subtyping is essential for accurate risk stratification [19,57].
Mixed	Shows features of both embryonal and alveolar types. Diagnosis depends on dominant histological and molecular characteristics; prognosis depends on fusion status [54].

**Table 2:** Common genes and proteins involved in the molecular pathogenesis of Wilms tumor (nephroblastoma). Genomic alterations include mutations, deletions, amplifications, and epigenetic dysregulation. Clinical associations are based on established relationships reported in the literature.

Gene	Function/Relevance	Genomic Location/Alteration	Additional Relationships
*WT1*	Tumor suppressor involved in kidney and genitourinary development; regulates differentiation and apoptosis	Chromosome 11p13 (deletion/mutation)	Most mutated gene in syndromic and sporadic WT; expressed in kidneys, gonads, and mesothelium [21,65].
*WT2* (11p15 region)	Imprinting region affecting growth-related genes such as *IGF2*; does not represent a single defined gene	Chromosome 11p15 (loss of imprinting or epigenetic dysregulation)	Associated with Beckwith-Wiedemann syndrome and sporadic WT; abnormal IGF2 expression promotes tumor growth [60].
*CTNNB1* (β-catenin)	Oncogene in Wnt signaling pathway; regulates cell adhesion and transcription/	Activating mutations	Mutations often co-occur with *WT1* alterations; seen in approximately 15% of WT cases [21].
*MYCN*	Oncogene that promotes cell proliferation and growth	Amplification	High-level amplification linked to poor prognosis and unfavorable histology [65].
*WTX* (AMER1)	Tumor suppressor involved in Wnt signaling regulation	X chromosome (Xq 11.1)	Inactivated in 20–30% of WT cases; more frequent in males [63].
*TP53*	Tumor suppressor; regulates cell cycle and apoptosis	Various mutations	Associated with anaplastic (unfavorable) histology and therapy resistance [63].
*IGF2*	Growth factor; promotes proliferation	Overexpression (due to 11p15 loss of imprinting)	Strongly expressed in tumors with 11p15 abnormalities; often silences *H19* [64].

## Data Availability

No new data were created or analyzed in this study.
